# Pharmacological effects of Bufei Jianpi granule on chronic obstructive pulmonary disease and its metabolism in rats

**DOI:** 10.3389/fphar.2022.1090345

**Published:** 2022-12-15

**Authors:** Xin-Xin Yang, Shuai Wang, Lin-Lin Cui, Tian-Jiao Li, Gang Bai, Yong-Rui Bao, Xian-Sheng Meng

**Affiliations:** ^1^ College of Pharmacy, Liaoning University of Traditional Chinese Medicine, Dalian, China; ^2^ Liaoning Multi-Dimensional Analysis of Traditional Chinese Medicine Technical Innovation Center, Dalian, China; ^3^ Liaoning Province Modern Chinese Medicine Research Engineering Laboratory, Dalian, China; ^4^ State Key Laboratory of Medicinal Chemical Biology, College of Pharmacy and Tianjin Key Laboratory of Molecular Drug Research, Nankai University, Tianjin, China

**Keywords:** Bufei Jianpi granule (BJG), chronic obstructive pulmonary disease (COPD), pharmacological effects, metabolites, UPLC-QTOF-MS/MS technology

## Abstract

This work was performed to determine the pharmacological effects of Bufei Jianpi granules on chronic obstructive pulmonary disease and its metabolism in rats.

Chronic obstructive pulmonary disease (COPD), ranked as the third leading cause of death worldwide, is seriously endangering human health. At present, the pathogenesis of COPD is complex and unclear, and the drug treatment mainly aims to alleviate and improve symptoms; however, they cannot achieve the purpose of eradicating the disease. Bufei Jianpi granule (BJG) is a Chinese medicine developed by the First Affiliated Hospital of Henan University of Traditional Chinese Medicine for treating COPD. This study focuses on the pharmacological effects of BJG on COPD and its metabolism in rats, aiming to provide a scientific basis for developing BJG against COPD. A total of 72 Sprague–Dawley (SD) rats were divided into the blank group, model group, positive control group, and BJG groups (2.36, 1.18, and 0.59 g/kg). Except for the blank group, rats in other groups were administered lipopolysaccharide (LPS) combined with smoking for 6 weeks to establish the COPD model. After another 6 weeks of treatment, the therapeutic effect of BJG on COPD rats was evaluated. In the BJG (2.36 g/kg) group, the cough condition of rats was significantly relieved and the body weight was close to that of the blank group. Compared with the mortality of 16.7% in the model group, no deaths occurred in the BJG (2.36 g/kg) and (1.18 g/kg) groups. The lung tissue damage in the BJG groups was less than that in the COPD group. Compared with the model group, MV, PIF, PEF, and EF50 in the BJG groups were observably increased in a dose-dependent manner, while sRaw, Raw, and FRC were obviously decreased. Also, the contents of IL-6, IL-8, TNF-α, PGE2, MMP-9, and NO in the serum and BALF were lowered dramatically in all BJG groups. All indicators present an obvious dose–effect relationship. On this basis, the UPLC-QTOF-MS/MS technology was used to analyze characteristic metabolites in rats under physiological and pathological conditions. A total of 17 prototype and 7 metabolite components were detected, and the concentration of most components was increased in the COPD pathologic state. It is suggested that BJG has a pharmacological effect in the treatment of COPD and the absorption and metabolism of chemical components of BJG in rats exhibited significant differences under physiological and pathological conditions.

## 1 Introduction

Chronic obstructive pulmonary disease (COPD) is a preventable and treatable lung disease characterized by continuous airflow restriction (Rong et al., 2020; Huang et al., 2022). Its airflow restriction mostly develops in a progressive manner, which is associated with the enhanced chronic inflammatory response of airways and lung tissue to harmful gases or particles such as tobacco smoke. It is a common and frequently occurring disease that seriously endangers human health (Ono et al., 2020). In 2020, it was ranked as the third leading cause of death in the world along with hypertension and diabetes, seriously endangering physical and mental health of humans (Song et al., 2021; Zou et al., 2022). The treatment of COPD mainly includes drug treatment and nondrug treatment, where the former is the key to improving the symptoms of COPD and reducing acute exacerbation (Li, 2020). Thus, long-term adherence to drug therapy and regular follow-up should be ensured. Furthermore, in terms of drug treatment, it mainly involves bronchodilators, glucocorticoids, and expectorants (Lin et al., 2020). However, due to the complex pathogenesis of COPD and incomplete understanding, drug treatment mainly aims to relieve and improve symptoms but is unable to achieve the goal of radical cure of the disease.

As an effective preparation commonly used in the First Affiliated Hospital of Henan University of Traditional Chinese Medicine for the treatment of COPD, the Bufei Jianpi granule (BJG) is composed of 12 traditional Chinese medicines (TCMs), including *Astragalus mongholicus* Bunge [Fabaceae; *Astragalus mongholicus* radix], *Codonopsis pilosula* (Franch.) Nannf [Campanulaceae; *Codonopsis pilosula* radix], *Polygonatum kingianum* Collett & Hemsl [Asparagaceae; *Polygonatum kingianum* rhizoma], *Atractylodes macrocephala* Koidz [Asteraceae; *Atractylodis macrocephalae* rhizoma], *Poria cocos* (Schw.) Wolf [Poromycelidae; *Poria*], *Fritillaria thunbergii* Miq [Liliaceae; *Fritillariae thunbergii* bulbus], *Pheretima aspergillum* (E. Perrier) [Lumbricidae; *Pheretima*], *Magnolia officinalis* Rehder & E. H. Wilson [Magnoliaceae; *Magnoliae officinalis* cortex], *Citrus reticulata* Blanco [Rutaceae; *Citri reticulatae* pericarpium], *Aster tataricus* L. f [Asteraceae; *Asteris* radix et rhizoma], *Epimedium brevicornu* Maxim [Berberidaceae; *Epimedii* folium], and *Ardisia japonica* (Thunb.) Blume [Primulaceae; *Ardisiae japonicae* herba] at a ratio of 12:6:12:9:9:6:9:6:9:6:6:15 (Li and Li, 2011). Clinical studies found that BJG can efficiently enhance the pulmonary function of patients with COPD in the stable stage, lower the number of acute exacerbations, and improve exercise endurance (Tang et al., 2019). However, at present, it is only used internally in the First Affiliated Hospital of Henan University of Traditional Chinese Medicine. In order to meet the needs of the majority of patients, it is urgently needed to develop BJG into a Chinese patent medicine preparation with a national brand for the treatment of COPD. For this purpose, systemic research has been carried out on the pharmacological effects, mechanism of action, and process and quality control of BJG in the treatment of COPD (Yu et al., 2019; Cui et al., 2020).

In this study, the commonly recognized pharmacological model of lipopolysaccharide (LPS) combined with the smoke-induced rat COPD model was used. The daily physiological state, lung function, lung tissue appearance and morphology, lung histopathological changes, and inflammatory factors in the serum and bronchoalveolar lavage fluid (BALF) were taken as the detection indicators to investigate the therapeutic effect of BJG at different doses on COPD from the perspective of pharmacological efficacy. On this basis, adopting the UPLC-QTOF-MS/MS technology combined with tandem mass spectrometry fragment ion information and comparison methods of the reference substance, the components of BJG absorbed into blood and their metabolites were analyzed under physiological and pathological conditions. The differences in chemical component metabolism in rats were compared so as to explore the effective chemical components of the prescription and the law of disease treatment, which provide the experimental basis for the development of BJG as a new drug for the treatment of COPD.

## 2 Materials and methods

### 2.1 Chemicals and reagents

MS-grade methanol and acetonitrile were purchased from Merck (Germany). MS-grade formic acid was bought from Thermo Fisher Technology Co., Ltd. (United States). Mullein isoflavone glucoside, naringin, hesperidin, ononin, epimedoside A, icariin, nobiletin, and tangeretin (purities > 98%) were all purchased from Sichuan Vicky Biotechnology Co., Ltd. (Chengdu, China). Betaine, adenosine, magnoflorine, peimine, peiminine, vanillin, hesperetin, wogonin, honokiol, magnolol, and linoleic acid (purities > 98%) were all acquired from Chengdu Pufei De Biotech Co., Ltd. (Chengdu, China). Lipopolysaccharide (LPS) was offered by Shanghai Sulaibao Biotechnology Co., Ltd. (Shanghai, China). Interleukin-8 (IL-8), interleukin-6 (IL-6), tumor necrosis factor α (TNF-α), prostaglandin E2 (PGE2), matrix metalloproteinase-9 (MMP-9), and nitric oxide (NO) testing kits were purchased from Shanghai Langton Biotechnology Co., Ltd. (Shanghai, China).

### 2.2 Preparation process of Bufei Jianpi granule

BJG was provided by the First Affiliated Hospital of Henan University of Traditional Chinese Medicine and produced by Jiangyin Tianjiang Pharmaceutical Co., Ltd. (batch no. 1905301, China). The preparation process of BJG is an industrial production process amplified proportionally according to the following single feeding amount: *Astragalus mongholicus* Bunge [Fabaceae; *Astragalus mongholicus* radix] 12 g, *Codonopsis pilosula* (Franch.) Nannf [Campanulaceae; *Codonopsis pilosula* radix] 6 g, *Polygonatum kingianum* Collett & Hemsl [Asparagaceae; *Polygonatum kingianum* rhizoma] 12 g, *Atractylodes macrocephala* Koidz [Asteraceae; *Atractylodis macrocephalae* rhizoma] 9 g, *Poria cocos* (Schw.) Wolf [Poromycelidae; *Poria*] 9 g, *Fritillaria thunbergii* Miq [Liliaceae; *Fritillariae thunbergii* bulbus] 6 g, *Pheretima aspergillum* (E. Perrier) [Lumbricidae; *Pheretima*] 9 g, *Magnolia officinalis* Rehder & E. H. Wilson [Magnoliaceae; *Magnolia officinalis* cortex] 6 g, *Citrus reticulata* Blanco [Rutaceae; *Citri reticulatae* pericarpium] 9 g, *Aster tataricus* L. f [Asteraceae; *Asteris* radix et rhizoma] 6 g, *Epimedium brevicornu* Maxim [Berberidaceae; *Epimedii* folium] 6 g, and *Ardisia japonica* (Thunb.) Blume [Primulaceae; *Ardisia japonicae* herba] 15 g.

After being crushed, *Atractylodis macrocephalae* rhizoma was combined with *Citri reticulatae* pericarpium, six times the volume of water was added, and the sample was hydrodistilled for 5 h. The volatile oil was collected. Then, the sample was filtered to get decoction (I), and the residue was collected for later use. Extract (I) was obtained as follows: *Magnolia officinalis* cortex, *Asteris* radix et rhizoma, and *Fritillariae thunbergii* bulbus are taken, six times the volume of 70% ethanol is added, the extract is refluxed for 1.5 h, and the process is repeated three times. The filtrates of three repetitions are combined, the sample is decompressed to recover ethanol, and it is concentrated to a relative density of 1.15 at 50°C to obtain extract (I). The residue was collected for later use. Extract (II) was obtained as follows: we combine the aforementioned drug residues, add eight times the volume of water, and decoct the sample three times for 1 h. Then, we filter and mix the filtrates with decoction (I), decompress the sample, and concentrate it to a relative density of 1.15 at 50°C to obtain extract (II). Dry powder (I) was obtained as follows: we combine extract (I) with extract (II) to obtain the total extract, 20% dextrin is added, and after spray-drying, dry powder (I) is obtained. Dry powder (II) was obtained as follows: the volatile oil is mixed with 10 times its amount of β-cyclodextrin by the grinding method to obtain the inclusion complex; after vacuum-drying and crushing, dry powder (II) is obtained. We mix dry powder (I), dry powder (II), and 10% honey of the total powder weight uniformly and make granules; after drying, the granules are formed (Li and Li, 2011).

### 2.3 Bufei Jianpi granule qualification

**FIGURE 1:**
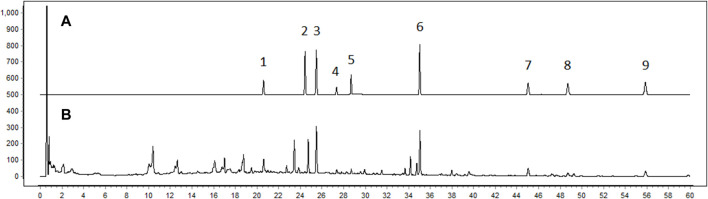
Fingerprint chromatogram of BJG. **(A)** Chromatogram of standard substances. 1: mullein isoflavone glucoside; 2: naringin; 3: hesperidin; 4: ononin; 5: epimedoside A; 6: icariin; 7: nobiletin; 8: tangeretin; 9: honokiol. **(B)** Chromatogram of the sample.

The quality of BJG was verified by HPLC detection (Figure 1). Mullein isoflavone glucoside, naringin, hesperidin, ononin, epimedoside A, icariin, nobiletin, tangeretin, and honokiol were quantified for controlling the quality of the granule, and their contents in the batch 1905301 sample were 0.071 mg/g, 0.172 mg/g, 0.028 mg/g, 0.017 mg/g, 0.021 mg/g, 0.343 mg/g, 0.047 mg/g, 0.018 mg/g, and 0.042 mg/g, respectively. The chemical composition of BJG was described in our previous study by the HPLC-QTOF-MS technology (Cui et al., 2020). Based on this, the authenticity of these nine quality control indicators was confirmed by comparing the retention time, accurate mass number, isotope peak, and other information of these chemical components (please refer to the Supplementary Material for details of HPLC and HPLC-QTOF-MS).

### 2.4 Animals

Male Sprague–Dawley (SD) rats (200 ± 20 g) were purchased from Liaoning Changsheng Biotechnology Co., Ltd. [number: SCXK (Liao) 2020-0001]. All rats were acclimated for 1 week after arrival and housed in a room under controlled temperature (22 ± 2°C) and humidity with a 12-h light/dark cycle and free access to water and food. Apart from that, all experiments were carried out following the approved animal protocols and guidelines established by the Medicine Ethics Review Committee for Animal Experiments of Liaoning University of Traditional Chinese Medicine with approval number 2020YS013(KT)-013-01.

### 2.5 Pharmacological efficacy of BJG on the COPD rat model

A total of 72 SD rats were randomly divided into six groups, namely, the blank group, COPD model group, AMINOPH (aminophylline) (0.0054 g/kg) positive control group, BJG high-dose (2.36 g/kg) group, medium-dose (1.18 g/kg) group, and low-dose (0.59 g/kg) group, with 12 rats in each group. The medium dose of BJG is determined according to the conversion of animal dose to human equivalent dose. For the high dose, we used two times the medium dose, and for low dose, we used two times below the medium dose, to investigate the dose–effect relationship. Except for the blank group, for rats in the other groups, the COPD model was established by administering LPS combined with smoking according to the method with minor modification (Shin et al., 2017; Mao et al., 2022; Pelgrim et al., 2022). The administration intervention was started in the 6th week, twice a day. The blank group and the model group were given distilled water of equal volume, and the whole process lasted for 6 weeks. During this period, the activity and behavior changes of the rats such as hair, mental state, activity ability, weight, death, and other physiological state indicators were observed and detected. After the drug intervention, the Buxco noninvasive animal airway detection system was used to measure the lung function of rats (Luo et al., 2021). The rats to be tested were placed into the noninvasive animal airway detection cavity. After the rats’ breathing was stable, the minute ventilation volume (MV), special airway resistance (sRaw), airway resistance (Raw), functional residual volume (FRC), peak inspiratory flow rate (PIF), peak expiratory flow rate (PEF), and expiratory flow rate at 50% ventilation (EF50) were measured continuously. In addition, the contents of IL-6, IL-8, TNF-α, PGE2, MMP-9, and NO in the serum and BALF were determined using ELISA kits.

### 2.6 Analysis of chemical components absorbed into the blood

A total of 24 SD rats were randomly classified into the blank group, blank administration group, model group, and model administration group. In the model groups, the rat model of COPD was established by administering LPS combined with the smoking method (Shin et al., 2017; Mao et al., 2022; Pelgrim et al., 2022), while in the administration groups, rats were gavaged with 2.36 g/kg solution of BJG, twice a day for 7 consecutive days. The blank group and the model group were given the same amount of normal saline. Then, 12 h before the last administration, fasting without water was performed, followed by 60 min after the last administration, blood being taken from the abdominal aorta into centrifuge tubes with heparin sodium, and 30 min later, it being centrifuged at 3,000 rpm for 15 min to separate the plasma. Afterward, the supernatant was taken and kept in a refrigerator at -80°C for later use.

### 2.7 Preparation of plasma samples

The plasma samples were thawed at room temperature, and 400 µl of plasma samples was accurately extracted, followed by protein removal with 1,200 µl of methanol. After that, by vortexing for 3 min and centrifugation at 13,000 rpm (4°C) for 10 min, the supernatant was taken, cryogenic freeze-dried, redissolved in 50 µl of methanol, and vortexed for 3 min. Furthermore, the supernatant was centrifuged again at 13,000 rpm (4°C) for 10 min and was directly detected by LC-MS (Li et al., 2022; Wang et al., 2022).

### 2.8 Chromatography–mass spectrometry analysis conditions

The positive ion mode is adjusted as follows: Agilent Poroshell 120 SB-C18 (100 mm × 4.6 mm, 2.7 μm) is used; the mobile phase consists of 0.1% formic acid water (A)–acetonitrile (B); gradient elution conditions are 0–25 min, 5–40% B; 25–35 min, 40–75% B; 35–40 min, and 75–100% B; the flow rate is 0.8 ml min^−1^; the column temperature is 30°C; the injection volume is 1 μl; the electrospray ion source (ESI) is detected in the positive ion mode; the drying gas flow is 13 L/min; the drying gas temperature is 350°C; the capillary voltage (Vcap) is 4,000 V; the neutralizer pressure is 45 psig; the fragmentor voltage is 125 V; the skimmer voltage is 65 V; the mass scanning range is 50–1,000 m/z; and the secondary MS collision voltage is 40 eV.

The negative ion mode is set as follows: Agilent Poroshell 120 SB-C18 (100 mm × 4.6 mm, 2.7 μm) is used; the mobile phase consists of water (A)–acetonitrile (B); gradient elution conditions are 0–30 min, 5–100% B; the flow rate is 0.8 ml min^−1^; the column temperature is 30°C; the injection volume is 5 μl; the drying gas flow is 11 L/min; the drying gas temperature is 250°C; the Vcap is 3,500 V; the neutralizer pressure is 45 psig; the fragmentor voltage is 125 V; the skimmer voltage is 65 V; the mass scanning range is 50–1,000 m/z; and the secondary MS collision voltage is 40 eV (Wang et al., 2021).

### 2.9 Data analysis

SPSS 19.0 software was adopted for statistical analysis. The data were represented as mean ± standard deviation (mean ± SD). Statistical comparisons were analyzed by one-way analysis of variance (ANOVA). A value of *p* < 0.05 suggests a difference, and *p* < 0.01 represents a significant difference (Chen et al., 2019; Liu et al., 2021).

MS data processing was performed with MassHunter software and the PCDL database, which contained extracted ion chromatograms and calculations of elemental compositions with mass errors within 10 ppm. The chemical structures of all analytes were explained and verified based on their elemental compositions, accurately measured mass values, the elution order on a C18 column, retention time, fragmentation behavior, and the comparison with authentic standards and literature as far as possible (Lu et al., 2021).

## 3 Results

### 3.1 General state of rats in the pharmacodynamic experiment

The rats in the blank group featured normal hair color and luster, good mental state, flexible and frequent activities, stable exhalation, and normal diet and water intake, while those in the model group had dull and yellow hair, no luster, cough, phlegm in the throat, mental fatigue, slow movement, reduced diet, and weight loss. In the BJG high-dose group and the AMINOPH group, cough was significantly relieved and phlegm and sound in the larynx disappeared, with daily activities of rats being more frequent. There was no significant difference in drinking water compared with the blank group. Furthermore, compared to the model group, the cough condition of rats in the BJG medium- and low-dose groups was reduced and their mental state was improved, while their water intake was reduced compared with the blank group.

### 3.2 Body weight change and deaths of rats

**FIGURE 2:**
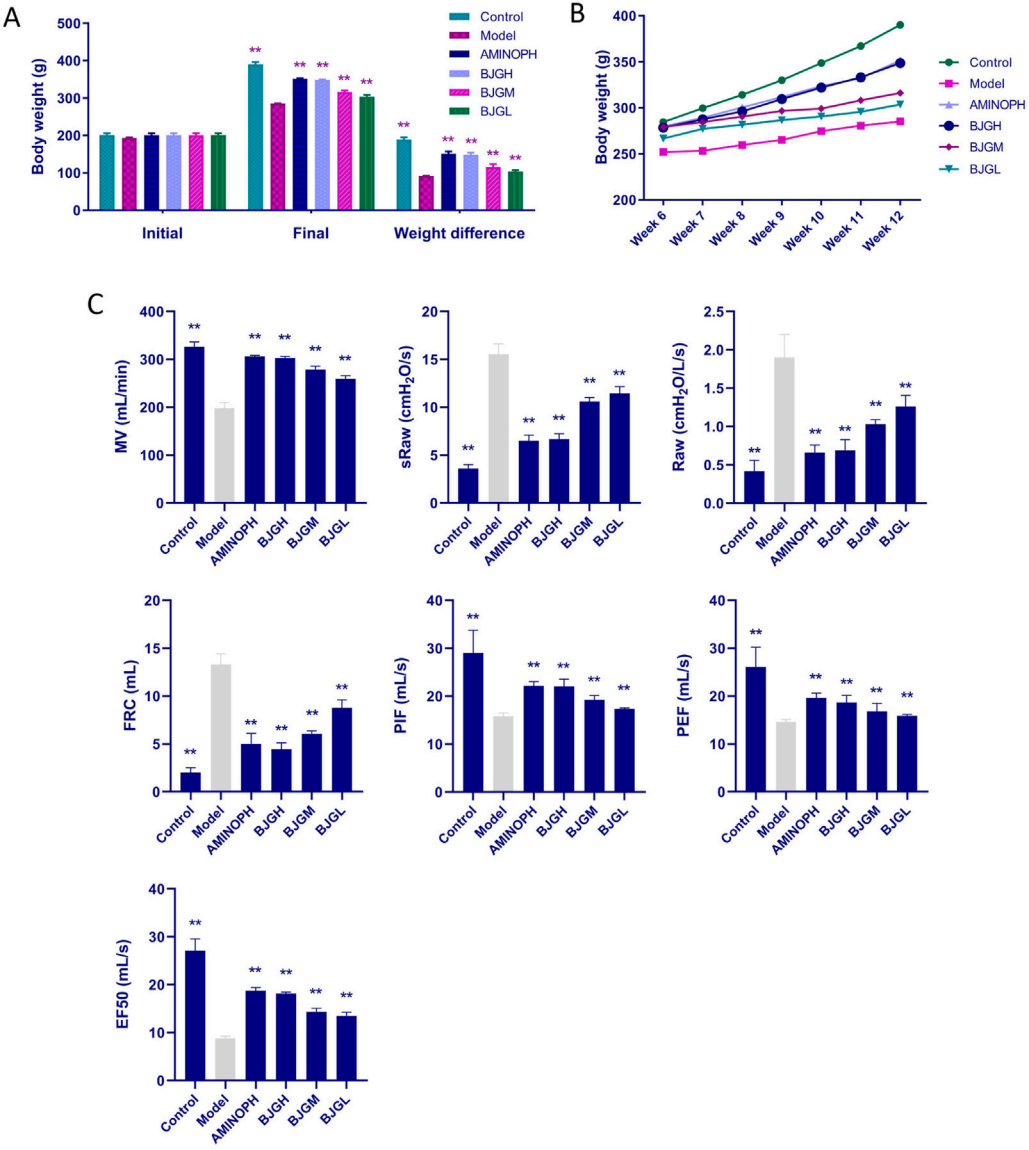
Changes in body weight and lung function of rats in each group. **(A)** Changes in body weight at the beginning and end of the experiment. **(B)** Changes in body weight of rats per week during treatment. **(C)** Changes in lung function indexes of rats in each group.

Before the experiment, there existed no significant difference in the weight of rats in each group. After the experiment, a significant difference in the body weight existed between the blank group and the model group (*p* < 0.01). In comparison with the model group, the body weight in the BJG groups and the AMINOPH group was obviously higher (*p* < 0.01) (Figure 2A). Before and after the experiment, the weight of rats in each group increased linearly. The weight growth rate of the blank group was faster, and that of the model group was the slowest (Figure 2B). Furthermore, during the whole experiment, there was no death in the blank control group and BJG high-dose, medium-dose, and AMINOPH groups, while deaths occurred in the model and BJG low-dose groups, with mortality rates of 16.7% and 8.3%, respectively.

### 3.3 Evaluation of lung function in rats

In comparison with the blank group, MV, PIF, PEF, and EF50 in the model group were dramatically decreased, while sRaw, Raw, and FRC were significantly increased (*p* < 0.01). Compared with the model group, MV, PIF, PEF, and EF50 in the BJG groups and the AMINOPH group were observably increased, while sRaw, Raw, and FRC were obviously decreased (*p* < 0.01) (Figure 2C). According to the results, compared with the blank group, the model group had increased airway resistance, decreased lung compliance, restricted airflow, and airway obstruction. However, compared with the model group, the indexes of each treatment group, especially the BJG high-dose and AMINOPH groups, had improved, indicating that BJG can ameliorate airway obstruction, enhance pulmonary ventilation, and exert a protective effect on the pulmonary function of COPD rats. In addition, a certain dose–effect relationship was observed.

### 3.4 Morphological changes in the lung tissue of rats

**FIGURE 3:**
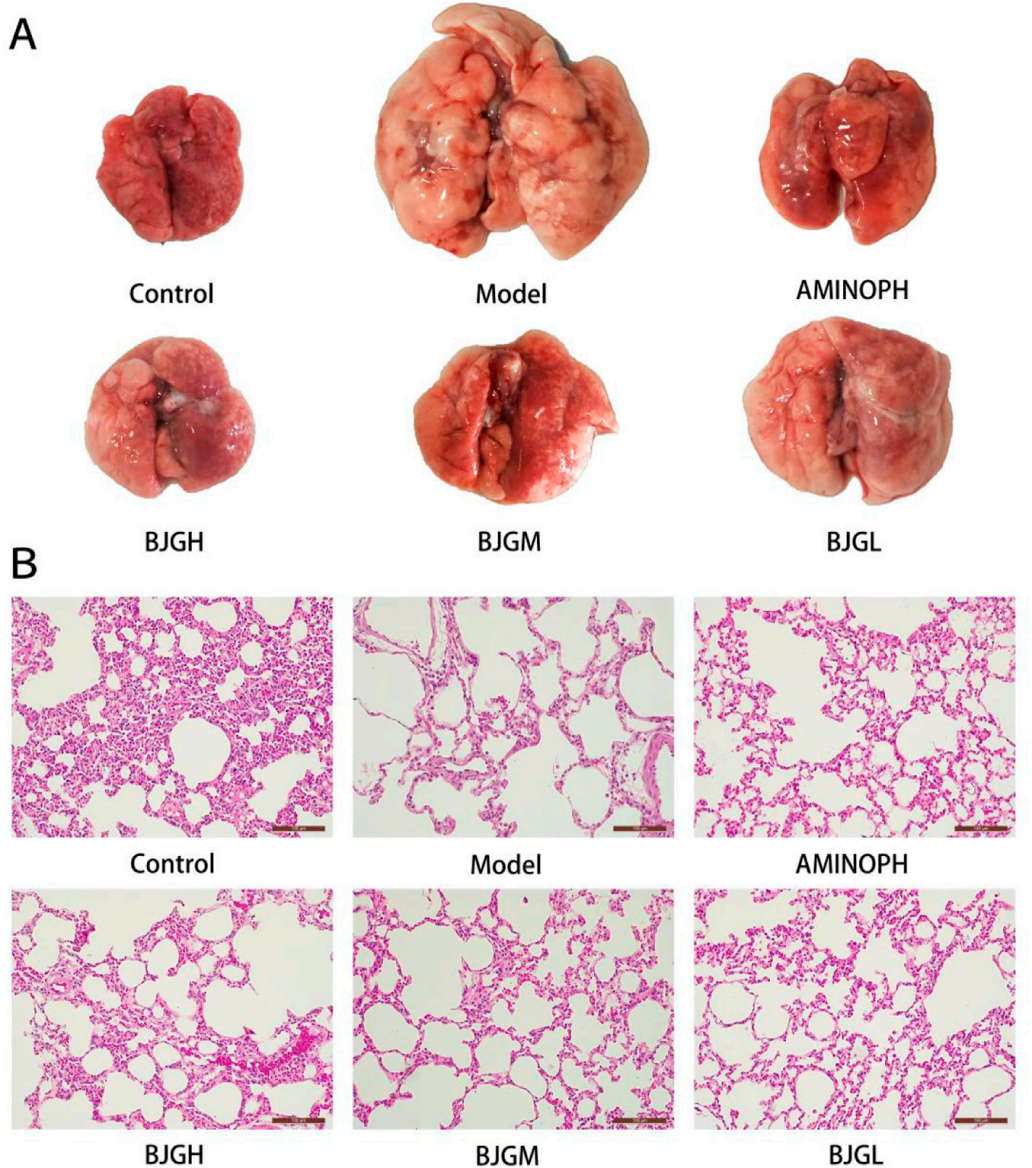
**(A)** Morphological changes in the lung tissue of rats. **(B)** Pathological changes in the lung tissue of rats [HE staining (magnification ×200)].

The lung tissues of the blank control group were smooth, shiny, soft, light red in color, and normal in size. In the model group, the surface of lung tissue was rough, the whole lung was white, luster was lost, and lung swelling was obvious. Compared with the model group, the lung volume of rats in each administration group was significantly reduced, shiny, and soft. The color of the BJG high-dose group and the AMINOPH group was close to that of the normal group, and the volume was reduced compared with the model group to a great extent. Moreover, the color of the BJG medium- and low-dose groups was lighter, and the volume was larger than that of the blank group (Figure 3A).

### 3.5 Pathological changes in the lung tissue of rats

Under the light microscope, the lung tissue structure of the blank group was complete and the bronchial tube wall was not thickened; other than that, there was no exudate in the lumen, the cilia were arranged neatly, and no inflammatory cell infiltration occurred. The size of the alveoli was normal, and the structure of the alveolar septum was clear. Compared with the blank group, the bronchial smooth muscle in the model group was significantly thickened and the lumen was filled with a large number of neutrophils. The alveolar septum was severely broken, alveolar cells were fibrotic, neutrophils were denatured and necrotic, cellulose was dissolved, alveolar walls were thinner, alveolar cavities were significantly expanded, and some alveoli fused to form pulmonary bullae. The pathological changes of the abovementioned trachea and lung tissues were consistent with those of COPD patients. Compared with the model group, the lung tissue damage in each drug intervention group was less than that in the COPD group and the lung tissue damage in the AMINOPH and BJG high-dose groups was the least (Figure 3B).

### 3.6 Changes in inflammatory factors in the rat serum and BALF

**FIGURE 4:**
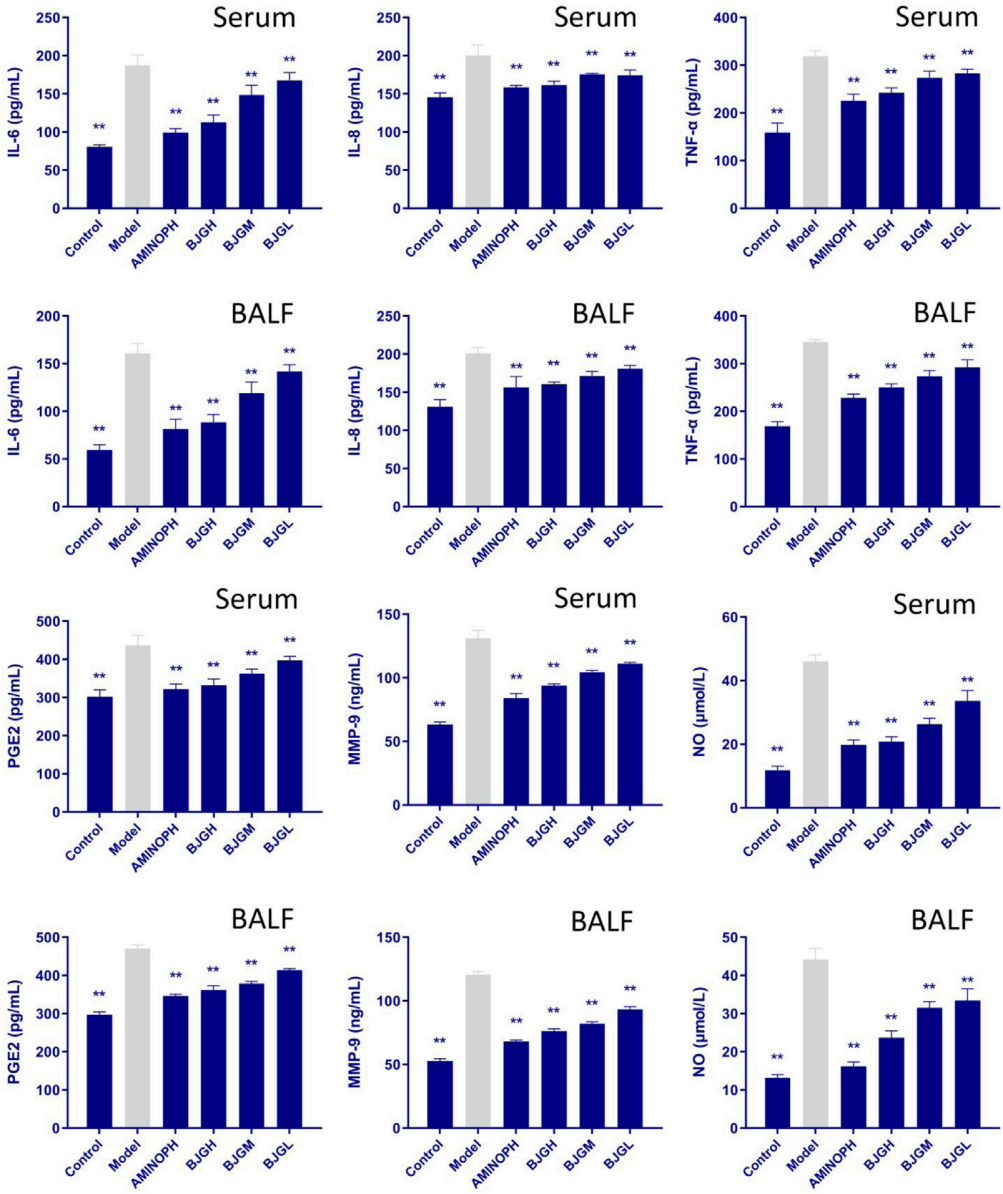
Changes in inflammatory factors in the rat serum and BALF.

The contents of IL-6, IL-8, TNF-α, PGE2, MMP-9, and NO in the serum and BALF of rats in each group are shown in Figure 4. Compared to the blank group, the expression levels of IL-6, IL-8, and TNF-α were increased in the model group and all treatment groups, and compared to the model group, they were lowered dramatically in all treatment groups (*p* < 0.01), especially in the BJG high-dose group and the AMINOPH group. Compared with the blank group, the expression levels of NO and MMP-9 factors in the model group and each treatment group were increased, while compared with the model group, they were decreased to a great extent in all treatment groups (*p* < 0.01), especially in the BJG high-dose group and the AMINOPH group. Moreover, compared with the blank group, the expression levels of PGE2 in the model group and each treatment group were increased, whereas compared with the model group, they were noticeably decreased in all treatment groups (*p* < 0.01), especially in the BJG high-dose group and the AMINOPH group. These findings suggest that all treatment groups could improve the inflammatory response in COPD rats.

### 3.7 LC-MS analysis on plasma samples of BJG under physiological and pathological conditions

**FIGURE 5:**
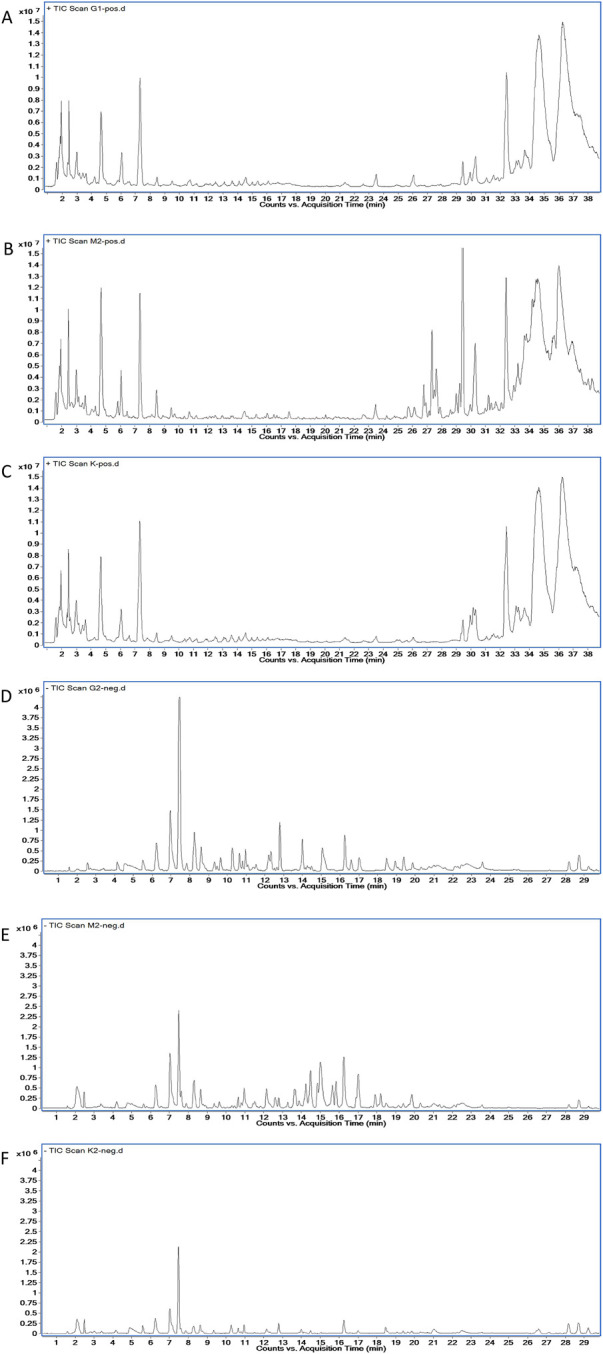
Plasma TIC of rats in each group. **(A)** Drug-containing plasma of healthy rats in the positive mode. **(B)** Drug-containing plasma of COPD rats in the positive mode. **(C)** Blank plasma of healthy rats in the positive mode. **(D)** Drug-containing plasma of healthy rats in the negative mode. **(E)** Drug-containing plasma of COPD rats in the negative mode. **(F)** Blank plasma of healthy rats in the negative mode.

By comparing the profiles of positive and negative blank plasma and drug-containing plasma, 13 different compounds, including 10 prototype components and three metabolites, were found in the pathological plasma in the positive ion mode. In physiological plasma, it contains all pathological components, but there is one prototypical component and one metabolite more than that in the pathological state. In the negative ion mode, eight different compounds, involving five prototypes and three metabolites, were discovered in the pathological plasma. In the physiological state, it contains all pathological components, but there is one more prototypical blood-entering component than that in the pathological state. The total ion-flow chromatogram (TIC) of each group is shown in Figure 5, while the retention time and molecular weight of each component are displayed in Table 1.

**TABLE 1:** Analysis on chemical components absorbed in healthy rat plasma.

Positive ion mode				Negative ion mode			
No.	t_*R*_ (min)	Excimer ion MS (m/z)	Source	No.	t_*R*_/(min)	Excimer ion MS (m/z)	Source
P1	1.829	118.0861	Prototype	N1	11.495	593.1872	Prototype
*P2	3.022	268.1040	Prototype	N2	14.287	301.0724	Prototype
P3	5.427	254.1387	Prototype	*N3	17.067	283.0619	Prototype
P4	11.245	342.1695	Prototype	N4	20.129	265.1250	Prototype
P5	15.924	428.3144	Prototype	N5	21.497	265.1248	Prototype
P6	16.551	432.3460	Prototype	N6	29.629	279.2328	Prototype
P7	16.934	430.3308	Prototype	NM1	2.537	167.0215	Metabolites
P8	17.553	430.3301	Prototype	NM2	6.666	273.0065	Metabolites
P9	19.934	432.3461	Prototype	NM3	15.175	259.1026	Metabolites
P10	21.570	414.3359	Prototype				
P11	22.379	414.3357	Prototype				
*PM1	3.820	169.0354	Metabolites				
PM2	8.417	349.1822	Metabolites				
PM3	11.981	565.2861	Metabolites				
PM4	15.796	461.1066	Metabolites				

*Components that were not detected in the pathological state.

### 3.8 Identification of chemical components absorbed in rat plasma

In the positive ion mode, for compound P1 (1.829 min, m/z 118.0861) (C_5_H_11_NO_2_, M + H)^+^, the secondary fragment ions were m/z 84.9595 (M + H-2OH)^+^ and m/z 58.0650 (M-C_2_H_3_O_2_)^+^. After comparison with the betaine reference substance, the fragment ion information was basically consistent with that of betaine and it was determined to be betaine. For compound P2 (3.022 min, m/z 268.1040) (C_10_H_13_N_5_O_4_, M + H)^+^, the secondary fragment ions were m/z 136.0616 (M + H-C_5_H_8_O_4_)^+^, m/z 119.0349 (M + H-C_5_H_8_O_4_-NH_3_)^+^, and m/z 94.0400 (M-C_5_H_8_O_4_-NH_3_-CN)^+^. After comparison with the adenosine control substance, the fragment ion information was in line with that of adenosine and, finally, it was identified as adenosine. For compound P3 (5.427 min, m/z 254.1387) (C_13_H_19_NO_4_+H)^+^, the secondary fragment ion was m/z 161.0580 (M + H-H_2_O-C_3_H_9_NO)^+^. In addition, after comparison with the standard database information and references, the fragment ion information is roughly consistent with that of codonopsinol B. It was speculated to be codonopsinol B. For compound P4 (11.245 min, m/z 342.1695) (C_20_H_24_NO_4_+H)^+^, the secondary fragment ions were m/z 297.1130 (C_20_H_24_NO_4_-CH_2_O-CH_3_)^+^, m/z 282.0884 (C_20_H_24_NO_4_-CH_2_O-2CH_3_)^+^, m/z 222.0670 (C_20_H_24_NO_4_-CH_2_O-2CH_3_-C_2_H_4_O_2_)^+^, and m/z 191.0851 (C_20_H_24_NO_4_-CH_2_O-2CH_3_-C_2_H_4_O_2_-CH_3_O)^+^. The fragment ion information was in accordance with that of magnoflorine, by comparison with that of magnoflorine, and it was detected as magnoflorine. For compound P5 (15.924 min, m/z 428.3139) (C_27_H_41_NO_3_+H)^+^, the secondary fragment ion was m/z 412.3205 (C_27_H_41_NO_3_+H-O)^+^. After comparison with the standard database information, the fragment ion information was roughly consistent with that of peimisine, so it was speculated that it might be peimisine. For compound P6 (16.551 min, m/z 432.3460) (C_27_H_45_NO_3_)^+^, the main secondary fragment ions were m/z 414.3358 (C_27_H_45_NO_3_-H_2_O)^+^, m/z 398.3054 (C_27_H_45_NO_3_-H_2_O-O)^+^, and m/z 299.2351 (C_27_H_45_NO_3_-H_2_O-O-C_6_H_11_O)^+^. After comparison with the control substance, the fragment ion information was basically in line with that of the control substance and the fragment ion information was determined to be peimine. In terms of compound P7 (16.934 min, m/z 430.3308) (C_27_H_43_NO_3_+H)^+^, the secondary fragment ion was m/z 412.3199 (C_27_H_43_NO_3_+H-H_2_O)^+^. After comparison with standard database information, fragment ion information was roughly consistent with that of zhebeinone, so it was speculated to be zhebeinone. As for compound P8 (17.553 min, m/z 430.3301) (C_27_H_43_NO_3_+H)^+^, the secondary fragment ions were m/z 412.3199 (C_27_H_43_NO_3_+H-H_2_O)^+^, m/z 396.2898 (C_27_H_43_NO_3_+H-H_2_O-O)^+^, and m/z 175.1474 (C_27_H_43_NO_3_+H-H_2_O-O-C_15_H_27_N)^+^. After comparison with the control substance, it was confirmed that it was peiminine. For compound P9 (19.934 min, m/z 432.3461) (C_27_H_45_NO_3_+H)^+^, the main secondary fragment ion is m/z 414.3359 (C_27_H_45_NO_3_+H-H_2_O)^+^. After comparison of the fragment ion information with the standard database, it was speculated to be zhebeinine. For compounds P10 (21.570 min, m/z 414.3359) and P11 (22.379 min, m/z 414.3357), (C_27_H_43_NO_2_+H)^+^ was compared with standard database information and fragment ion information, and it was speculated to be ebeiedinone or zhebeirine. The specific association needs to be further confirmed by a reference substance. Compound PM1 with molecular ion peak m/z 169.0354 (C_5_H_4_N_4_O_3_+H)^+^, which is 32 Da more than the hypoxanthine excimer ion peak 137 (C_5_H_4_N_4_O + H)^+^, prompted that O is added continuously and two oxygenation reactions occurred. Combined with the fragments m/z 152.0319 (C_5_H_4_N_4_O_3_+H-NH_3_)^+^, m/z 141.0669 (C_5_H_4_N_4_O_3_+H-CO)^+^, and m/z 125.9855 (C_5_H_4_N_4_O_3_+H-CONH)^+^, it is speculated to be hypoxanthine oxidation products. Compound PM2 m/z 349.1822 (C_16_H_12_O_7_S + H)^+^, which was found to be 80 Da more than the formononetin excimer ion peak 269 (C_16_H_12_O_4_+H)^+^, suggested that the compound is a sulfate metabolite. Combined with fragment m/z 269.1024 (C_16_H_12_O_4_+H)^+^, which is the prototype component of formononetin, it was speculated that the combination is formononetin sulfate. Furthermore, for compound PM3 with the molecular ion peak of m/z 565.2861, the main secondary fragment ion is m/z 403.1357 (C_21_H_22_O_8_+H)^+^, which is the excimer ion peak of nobiletin. The molecular weight of this substance in primary mass spectrometry is 162 Da more than that of nobiletin. It may be the product of glucuronidation and demethylation of nobiletin. Compound PM4 with m/z 461.1066, which is 176 Da more than that of calycosin excimer ion peak 285, demonstrated that the compound is a metabolite of glucoaldehyde acidification and the corresponding molecular formula is C_22_H_20_O_11_. Combined with fragments m/z 285.0753 (C_16_H_12_O_5_+H)^+^, m/z 270.0509 (C_16_H_12_O_5_+H-CH_3_)^+^, and m/z 125.9863 (C_16_H_12_O_5_+H-C_9_H_4_O_3_)^+^, m/z 285.0753 is the prototype component of the compound calycosin, so that the compound is speculated to be glucuronized calycosin.

In the negative ion mode, for compound N1 (11.495 min, m/z 593.1872) (C_28_H_34_O_14_-H)^-^, the secondary fragment ions were m/z 510.2635 (C_28_H_34_O_14_-C_4_H_4_O_2_)^-^, m/z 285.0783 (C_28_H_34_O_14_-C_12_H_21_O_7_)^-^, and m/z 96.9587 (C_28_H_34_O_14_-C_20_H_25_O_12_)^-^. After comparison with vanillin, the fragment ion information was basically consistent and it was identified as vanillin. For compound N2 (14.287 min, m/z 301.0724) (C_28_H_34_O_14_-H)^-^, the main secondary fragment ions were m/z 136.0171 (C_28_H_34_O_14_-C_20_H_26_O_12_)^-^, m/z 108.0223 (C_28_H_34_O_14_-C_21_H_26_O_13_)^-^, and m/z 65.0039 (C_28_H_34_O_14_-C_23_H_29_O_14_)^-^. After comparison with hesperetin, the fragment ion information was consistent with that of hesperetin. Therefore, it was identified as hesperetin. For compound N3 (17.067 min, m/z 283.0619) (C_16_H_12_O_5_-H)^-^, the secondary fragment ions were m/z 239.0344 (C_16_H_12_O_5_-C_2_H_5_O)^-^, m/z 163.0039 (C_16_H_12_O_5_-C_7_H_5_O_2_)^-^, m/z 135.0099 (C_16_H_12_O_5_-C_7_H_5_O_2_-CO)^-^, and m/z 110.0007 (C_16_H_12_O_5_-C_7_H_5_O_2_-CO-C_2_H_5_)^-^. After comparison with the control substance, the fragment ion information was in line with that of wogonin. It was finally determined to be wogonin. For compound N4 (20.129 min, m/z 265.1250) (C_18_H_18_O_2_-H)^-^, the secondary fragment ions were m/z 249.0936 (C_18_H_18_O_2_-OH)^-^, m/z 223.0771 (C_18_H_18_O_2_-C_3_H_7_O)^-^, and m/z 197.0617 (C_18_H_18_O_2_-C_4_H_5_O_2_)^-^. After comparison with the honokiol control substance, fragment ion information was basically consistent with that of honokiol and it was identified as honokiol. For compound N5 (21.576 min, m/z 265.1248) (C_18_H_18_O_2_-H)^-^, the main secondary fragment ions were m/z 223.0767 (C_18_H_18_O_2_-H-C_3_H_5_)^-^, m/z 204.0577 (C_18_H_18_O_2_-H-C_3_H_5_-OH)^-^, and m/z 119.0501 (C_18_H_18_O_2_-C_9_H_7_O_2_)^-^. After comparison with the reference substance, it was identified as magnolol. For compound N6 (29.296 min, m/z 279.2328) (C_18_H_32_O_2_-H)^-^, no fragment ions were produced at 40 V. After comparison with the linoleic acid reference substance, it was determined to be linoleic acid. The molecular ion peak of compound NM1 was m/z 167.0215, which is the methyl group, 14 Da more than protocatechuic acid. When combined with the fragment m/z 122.4525 (C_7_H_6_O_4_-2O)^-^ and according to the literature (Guo et al., 2019) as well as database comparison, it was speculated as a methylated product of protocatechuic acid. For compound NM2 with m/z 273.0065, which increased by 94 Da (M + CH_2_+SO_3_) compared with caffeic acid (C_9_H_8_O_4_), according to the comparison of literature studies and databases, it was speculated that the compound was 3-methoxy-caffeine-4-O-sulfate. Moreover, the basic ion peak of compound NM3 was m/z 259.1026, 80 Da more than that of caffeic acid 179, suggesting that M + SO_3_ was increased. Beyond that, the corresponding molecular formula was C_9_H_8_O_7_S, and in comparison with the characteristic ion of caffeic acid with m/z 179.0346, it was speculated to be caffeic acid-3-O-sulfate.

### 3.9 Difference of prototype components from BJG under physiological and COPD pathological conditions

**FIGURE 6:**
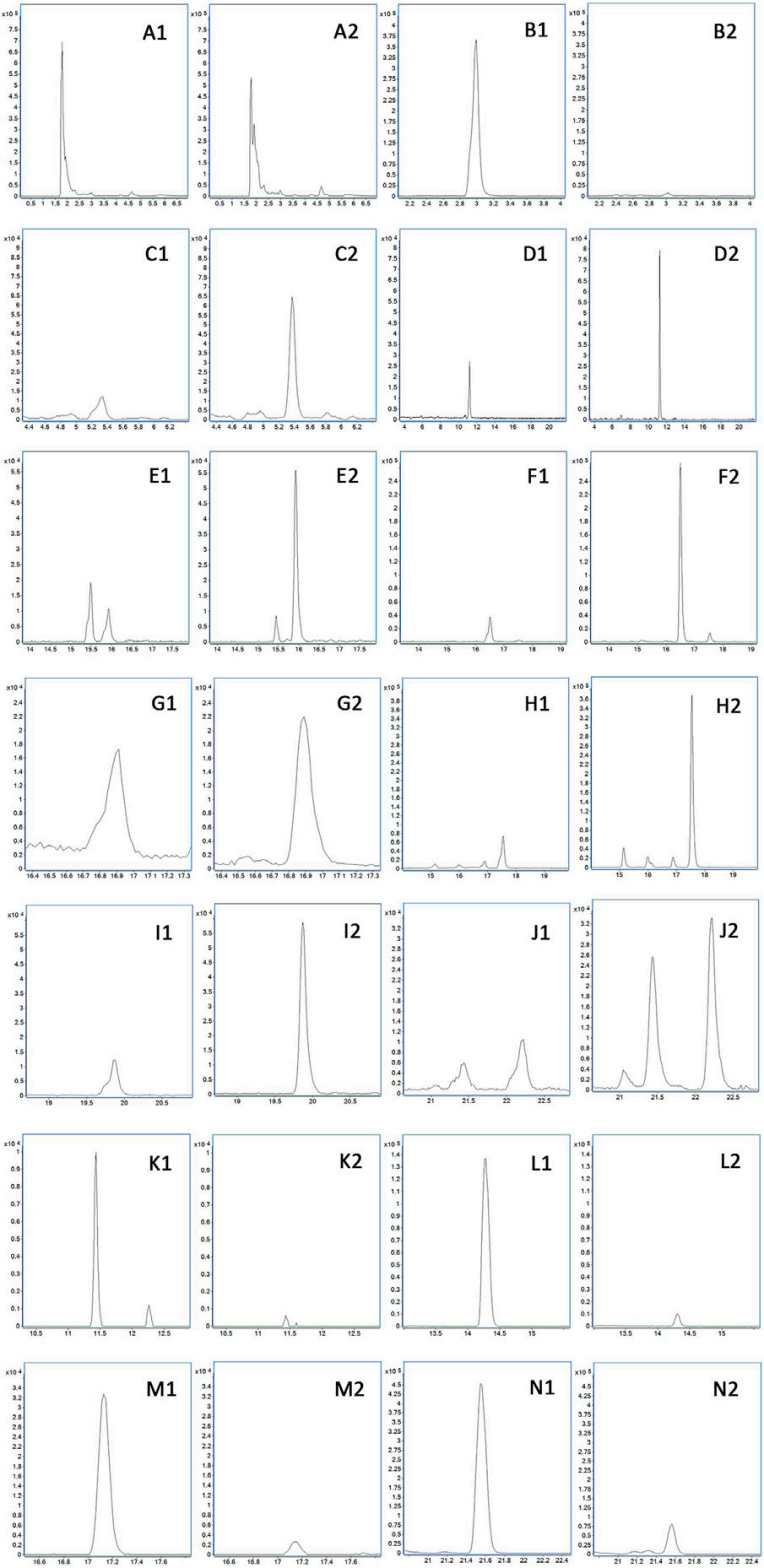
Difference of prototype blood components. **(A)** Betaine. **(B)** Adenosine. **(C)** Codonopsinol B. **(D)** Magnoflorine. **(E)** Peimisine. **(F)** Peimine. **(G)** Zhebeinone. **(H)** Peiminine. **(I)** Zhebeinine. **(J)** Ebeiedinone and Zhebeirine. **(K)** Vanillin. **(L)** Hesperetin. **(M)** Wogonin. **(N)** Magnolol. 1 presents the physiological state, and 2 presents the pathological state.

According to Table 2 and Figure 6, betaine, codonopsinol B, magnoflorine, peimisine, peimine, zhebeinone, peiminine, zhebeinine, ebeiedinone, zhebeirine, vanillin, hesperetin, honokiol, magnolol, and linoleic acid were all absorbed into the blood as the prototype under physiological and pathological conditions. Taking the variation of abundance greater than 1.5 times as the statistical standard, nine of the prototype components increased in the blood, five decreased, and one was not detected under the pathological state, while honokiol and linoleic acid did not change.

**TABLE 2:** Difference of prototype blood components.

No.	Compound	Physiological state	Relative abundance of the physiological state	Pathological state	Relative abundance of the pathological state
1	Betaine	√	1	√	↓
2	Adenosine	√	1	-	0
3	Codonopsinol B	√	1	√	↑
4	Magnoflorine	√	1	√	↑
5	Peimisine	√	1	√	↑
6	Peimine	√	1	√	↑
7	Zhebeinone	√	1	√	↑
8	Peiminine	√	1	√	↑
9	Zhebeinine	√	1	√	↑
10	Ebeiedinone/zhebeirine	√	1	√	↑
11	Ebeiedinone/zhebeirine	√	1	√	↑
12	Vanillin	√	1	√	↓
13	Hesperetin	√	1	√	↓
14	Wogonin	√	1	√	↓
15	Honokiol	√	1	√	-
16	Magnolol	√	1	√	↓
17	Linoleic acid	√	1	√	-

### 3.10 Difference of metabolites from BJG under physiological and COPD pathological conditions

**FIGURE 7:**
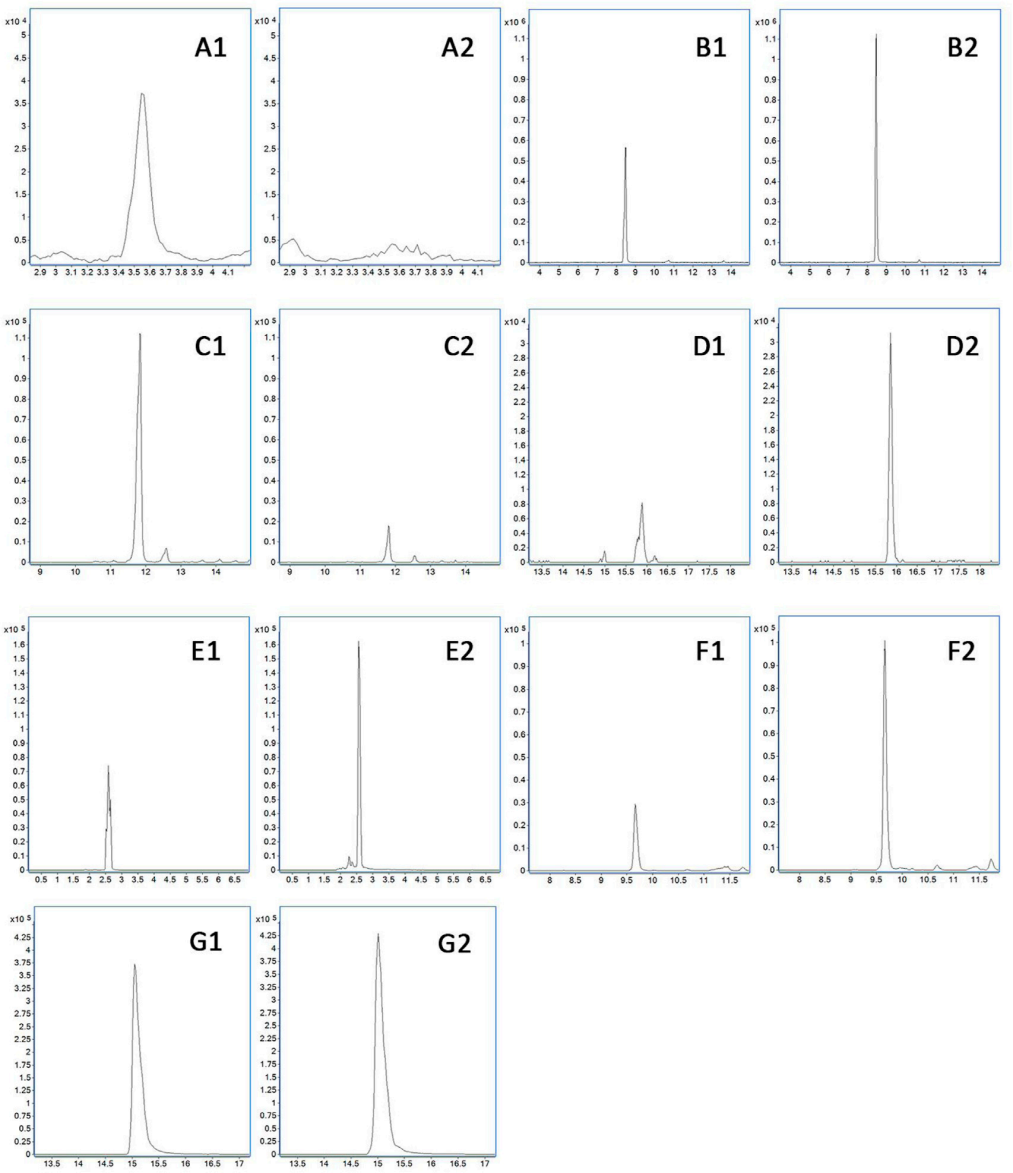
Difference of metabolites in the blood. **(A)** Hypoxanthine oxidation products. **(B)** Formononetin sulfate. **(C)** Nobiletin glucuronidation and demethylation product. **(D)** Glucuronized calycosin. **(E)** Methylated product of protocatechuic acid. **(F)** 3-Methoxy-caffeine-4-O-sulfate. **(G)** Caffeic acid-3-O-sulfate. 1 presents the physiological state, and 2 presents the pathological state.

It can be seen from Table 3 and Figure 7 that there are seven metabolic components that change under the pathological state compared with the physiological state. Among them, five components increase in the blood concentration under the pathological state, while one metabolite decreases in the blood concentration and hypoxanthine oxidation products are not detected under the pathological state, which may not affect the absorption of the aforementioned components under the pathological state.

**TABLE 3:** Difference of metabolites in the blood.

No.	Compound	Physiological state	Relative abundance of the physiological state	Pathological state	Relative abundance of the pathological state
1	Hypoxanthine oxidation products	√	1	-	0
2	Formononetin sulfate	√	1	√	↑
3	Nobiletin glucuronidation and demethylation product	√	1	√	↓
4	Glucuronized calycosin	√	1	√	↑
5	Methylated product of protocatechuic acid	√	1	√	↑
6	3-Methoxy-caffeine-4-O-sulfate	√	1	√	↑
7	Caffeic acid-3-O-sulfate	√	1	√	↑

## 4 Discussion

COPD is a preventable and treatable lung disease characterized by a persistent and progressive limitation of airflow (Rong et al., 2020; Huang et al., 2022). Its airflow restriction is associated with the enhanced chronic inflammatory response of the airways and lung tissue to harmful gases or particles such as tobacco smoke (Ono et al., 2020). COPD is a common and frequently occurring disease that poses a serious threat to human health (Li et al., 2020). The LPS combined with the smoke-induced rat COPD model is a classical and commonly used model to evaluate the drug treatment of COPD, based on which this study investigated the therapeutic effect of BJG on COPD from physiological, pathological, biochemical, and other aspects, providing better experimental data for its development into a new clinical drug for COPD.

The mental state directly reflects the physiological or physical state. In this study, the rats in the COPD model group were listless and sluggish, with dull and yellow hair and cough and throat phlegm sounds. Patients with advanced COPD tend to lose weight (Pershina et al., 2019), while rats in the model group presented an emaciated body type, and death occurs with a mortality rate of 16.7%. After the BJG treatment with different dosages, the state of the rats was improved by different degrees. In particular, the rats in the BJG high-dose group had more frequent daily activities, and the phlegm sounds between the larynx disappeared. There was no difference in weight and water intake between the BJG high-dose and blank groups, and no deaths occurred.

Airway resistance refers to the viscous resistance generated by airflow, which is caused by the friction between airflow and the airway wall as well as by airflow itself when air flows through the airway during breathing. COPD is characterized by airflow restriction that is not completely reversible. Pulmonary function examination is the main objective index to judge airflow restriction, and it is an important index to diagnose COPD and evaluate the therapeutic effect (Kilk et al., 2018; Liu et al., 2018). Airway resistance is inversely related to the lung volume. In this study, sRaw, Raw, FRC, and other pulmonary function indicators reflecting airway resistance were significantly increased in the model group, while MV, PIF, PEF, and EF50 were significantly decreased, indicating that the COPD model was successfully established. The primary therapy for COPD is intended to reduce airway resistance (James et al., 2019). All doses of BJG treatment groups can significantly improve the phenomenon of increased airway resistance, decreased pulmonary compliance, airflow restriction, and airway obstruction in COPD rats, especially in the high-dose group. These results indicated that BJG possesses protective effects on lung function in COPD rats and exerts a certain dose–effect relationship.

The main pathological changes of COPD are manifested by chronic bronchitis and emphysema (Jia et al., 2020). In this study, the lungs of rats in the model group showed obvious histopathological changes. Increased lung size, dark color, and scattered foci of necrosis were observed. Also, the number of pulmonary alveoli decreased significantly, with damaged structure, irregular expansion, and thinner walls. The alveolar space narrowed, and some of it broke and fused to form large vesicles, thus forming emphysema. In addition, there are a lot of inflammatory cells infiltrating the pulmonary interstitium, alveolar cavity, and vascular cavity and the lung tissue presents obvious pathological damage. The damage of lung tissue in each dose group of BJG was less severe than that in the COPD group. No emphysema was formed, only a few inflammatory cells were infiltrated, and the damage degree of lung tissue in the BJG high-dose group was the least.

The results of inflammatory factor detection in the serum and BALF of rats in each group showed that the levels of IL-6, IL-8, and TNF-α in the model group were significantly increased and the contents of NO and MMP-9 reflecting oxidative damage and tissue damage were also significantly increased as well as those of PGE2, reflecting vasodilatation and vascular permeability. The expression levels of the aforementioned factors were significantly reduced in different doses of BJG groups, manifesting that BJG can improve the vasodilation and vascular permeability of COPD rats, improve the inflammatory reaction, and reduce the oxidative damage and tissue damage of COPD rats in a dose–effect relationship.

For proving that BJG possesses an established therapeutic effect on COPD, the UPLC-QTOF-MS/MS technology was adopted to analyze the differences in chemical component metabolism in rats under physiological and pathological conditions so as to explore the effective chemical components of the prescription and the law of disease treatment. Based on the preliminary study on the chemical components of 95 monomers in BJG (Cui et al., 2020), 17 prototype monomers, namely, betaine, adenosine, codonopsinol B, magnoflorine, peimisine, peimine, zhebeinone, peiminine, zhebeinine, ebeiedinone, zhebeirine, vanillin, hesperetin, wogonin, honokiol, magnolol, and linoleic acid, were identified in the blood component of BJG. Among them, 11 were discovered in the comparison of reference materials and six in the secondary comparison; in addition, seven metabolites, namely, hypoxanthine oxidation products, sulfated formononetin, glucoaldehyde acidification and demethylation of citrine, glucoaldehyde acidification of calycoflavone, protocatechuic acid methylation, 3-methoxy-caffeine-4-O-sulfate, and caffeine-3-O-sulfate, were confirmed in blood components. The products were derived from hypoxanthine, formononetin, citrine, calycosin, protocatechuic acid, and caffeic acid. Through this experiment, the research group found the tracks of 23 components in BJG in plasma and preliminarily clarified the blood transfer components and metabolism rules of the compound from *in vitro* to *in vivo*.

Through the comparative study on the blood components of BJG in physiological and COPD pathological states, it was found that 15 prototype components were detected in both physiological and pathological states. Among them, nine components increased in blood concentration in the pathological state and six prototype components decreased in blood concentration. Apart from that, seven metabolic components were detected under physiological and pathological conditions. Among them, five components increased in blood concentration and two components decreased in blood concentration. The aforementioned results show that the absorption of most chemical components is significantly enhanced when BJG is administered in the pathological state compared to that in the physiological state, and the structural types of these enhanced chemical components are mostly alkaloids and fatty acids, while the absorption of flavonoids and lignin is weakened. At the same time, it can be seen from the metabolites that the reason for the weakening of flavonoids and lignin components is related to the acceleration of compound metabolism in the pathological state, which leads to the decrease in peak time of components and the decrease in content at the same time. Furthermore, it is found that the physiological components include two more than the pathological prototype blood-entering components, namely, adenosine and wogonin, and the physiological components include one more than the pathological metabolites, which is hypoxanthine oxidation products. The reason is that, under pathological conditions, either the body performs poor absorption of this compound or all of it is converted into related metabolites after absorption.

By comparing the differences in blood-entering components of the compound under pathological and physiological conditions, it is observed that there are significant differences in the metabolism of chemical components in BJG in rats under pathological and physiological models. The absorption of alkaloids and fatty acids in BJG under pathological conditions is enhanced, while the absorption of flavonoids and lignin is weakened. At the same time, it also objectively reflects the difference between taking drugs with disease and taking drugs without disease in traditional medicine. Thus, it provides data and theoretical support for the study on the metabolic mechanism of traditional Chinese medicine in different states.

Moreover, the chemical components absorbed in rat plasma are related to acute lung injury, asthma, and pulmonary fibrosis to varying degrees. There also exists a deeper relationship between these active components and pharmacodynamic indicators. Among the alkaloids, peimine and peiminine can alleviate LPS- or bleomycin-induced acute lung injury in rats. They can inhibit lung inflammation and pulmonary fibrosis, resulting in their apparent inhibition of inflammatory factors such as TNF-α, IL-6, IL-1β, and IL-17 (Du et al., 2020; Liu et al., 2022). Betaine possesses anti-inflammatory activity, and it can reduce the inflammatory factors of TNF-α and IL-6 in the body (Detopoulou et al., 2008; Vinke et al., 2019). Ebeiedinone possesses a tracheobronchial relaxation effect (Wu et al., 2018). Most flavonoids possess potent anti-inflammatory activity, and some show antioxidant activity. For example, hesperetin can significantly reduce LPS-induced lung pathological injury and reduce the number of neutrophils and the levels of inflammatory cytokines TNF-α and IL-6 *in vivo* and *in vitro*. Also, the effect is related to regulating the TLR4-MyD88-NF-κB signaling pathway, affecting the miR-410/SOX18 axis, and targeting the MD2 protein (Wang et al., 2019; Ye et al., 2019; Dong et al., 2020). Wogonin can prevent LPS-induced acute lung injury and inflammation and inhibit the production of inflammatory cytokines such as TNF-α, IL-1β, and IL-6. The mechanism is due to the regulation of the PPARγ-involved NF-κB pathway and the reduction of p38 MAPK and JNK phosphorylation (Yao et al., 2014; Wei et al., 2017). Formononetin has significant anti-inflammatory and antioxidant effects in a variety of diseases. In the study on treatment of asthma, it can diminish the expression of IL-4, IL-5, IL-13, IL-17A, IgE, CCL5, and CCL11, inhibit NF-kB- and JNK-mediated inflammatory signaling, and reduce oxidative damage through decreasing the activity of ROS and increasing SOD (Yi et al., 2020). Calycosin has anti-inflammatory and antioxidant properties. It can obviously reduce the pathological damage to lung tissue and pulmonary edema in rats. The mechanism is through inactivating the HMGB1/MyD88/NF-κB pathway and the NLRP3 inflammasome (Chen et al., 2021). Moreover, nobiletin and vanillin also exert anti-inflammatory activity (He et al., 2019; Costantini et al., 2021). Lignans are also related to anti-inflammatory and antioxidant properties. Honokiol can inhibit TGF-β/Smad signaling, matrix proteins, and the IL-6/CD44/STAT3 axis to reduce pulmonary fibrosis, which exerts anti-inflammatory and antioxidant effects (Pulivendala et al., 2020). Magnolol represents excellent protective effects on LPS-induced acute lung injury in rats. It can relieve lung tissue damage and downregulate the levels of proinflammatory factors such as TNF-α, IL-1β and IL-6, which is related to PPAR-γ-dependent inhibition of NF-kB activation (Ni et al., 2012; Lin et al., 2015). Furthermore, caffeic acid can decrease the suppression of TNF-α, NF-κB, ERK1/2, STAT3, and JNK1/2 to play an anti-inflammatory role (Santos et al., 2019). Protocatechuic acid also possesses anti-inflammatory activity. Moreover, it can inhibit the inactivation of Smad2/3 proteins to exert the effect of inhibiting asthma airway remodeling (Lende et al., 2011; Liu et al., 2019).

In summary, as part of the research on the National Key R&D Program of China, this study proved that BJG is positively effective for the treatment of COPD and revealed the active components and their metabolism, which provide reliable pharmacodynamic data for developing BJG into a Chinese patent medicine preparation with a national brand for the treatment of COPD. To achieve this, further research will be carried out around the mechanism of BJG curing COPD and evidence-based medicine.

## 5 Conclusion

BJG can improve the survival status of COPD rats, improve lung function, reduce lung injury, vasodilation, and vascular permeability, improve inflammatory reactions, reduce oxidative damage and tissue damage, and possess a superior protective effect on the lung function of COPD rats. There are significant differences in the metabolism of chemical components of BJG in rats under pathological and COPD physiological states. Under pathological conditions, the absorption of alkaloids and fatty acids in BJG is enhanced, while the absorption of flavonoids and lignin is weakened. The aforementioned research provides a scientific basis for the development of BJG as a new clinical drug for the treatment of COPD.

## Data Availability

The original contributions presented in the study are included in the article/Supplementary Materials, further inquiries can be directed to the corresponding authors.

## Abbreviations

AMINOPH, aminophylline; BALF, bronchoalveolar lavage fluid; BJG, Bufei Jianpi granule; BJGH, Bufei Jianpi granule high-dose group; BJGM, Bufei Jianpi granule medium-dose group; BJGL, Bufei Jianpi granule low-dose group; COPD, chronic obstructive pulmonary disease; EF50, expiratory flow rate at 50% ventilation; ESI, electrospray ion source; FRC, functional residual volume; IL-8, interleukin-8; IL-6, interleukin-6; LPS, lipopolysaccharide; MMP-9, matrix metalloproteinase-9; MV, minute ventilation volume; NO, nitric oxide; PEF, peak expiratory flow rate; PGE2, prostaglandin E2; PIF, peak inspiratory flow rate; Raw, airway resistance; SD, Sprague–Dawley; sRaw, special airway resistance; TCMs, traditional Chinese medicines; TIC, total ion-flow chromatogram; TNF-α, tumor necrosis factor α; Vcap, capillary voltage.
