# Supplementary material for: Pharmacological effects of Bufei Jianpi granule on chronic obstructive pulmonary disease and its metabolism in rats
Source: Front Pharmacol. 2022 Dec 15;13:1090345. doi: 10.3389/fphar.2022.1090345 (PMC9797594; doi:10.3389/fphar.2022.1090345)
Supplement: Supplementary file 1 [file DataSheet1.docx]

Supplementary Material

# 1 Identification of 9 chemical constituents in BJG by HPLC-QTOF-MS

# 1.1 Sample preparation

10.0 g powder of BJG was accurately weighed and combined with 25 ml of methanol and ultrasonically extracted for 30 min, and then filtered.

**1.2 Methods**

An Agilent 1290 infinity HPLC system coupled with HPLC-QTOF-MS system was used (Agilent Technologies, Santa Clara, CA, USA). Agilent Poroshell 120 EC-C18 (100 mm×3.0 mm, 2.7 μm); mobile phase: 0.1% formic acid water (A)-acetonitrile (B); Gradient elution conditions: 0-5 min, 3-3% B; 5-60 min, 3-48% B; flow rate: 0.6 ml·min^-1^; column temperature: 40℃; injection volume: 5 μl. The electrospray ion source (ESI) is detected in the positive and negative ion mode; drying gas flow is 13 L/min, and drying gas temperature is 350℃, while the capillary voltage (Vcap) is 4000 V, Neulizerpressure 45 psig, Fragmentor 125 V, Skimmer 65 V, mass scanning range m/z 50-1500.

**1.3 Results**

The authenticity of mullein isoflavone glucoside, naringin, hesperidin, ononin, epimedoside A, icariin, nobiletin, tangeretin and honokiol in BJG was confirmed by comparing the retention time, accurate mass number, isotope peak and other information of these chemical components with standard substances. The mass spectrum bar graphs were shown in Supplementary Figure 1.

A2

A1

B1

B2

C2

C1

D2

D1

E2

E1

F2

F1

G2

G1

H2

H1

I2

I1

**Supplementary Figure 1.** MS chromatograms of the components. 1 represents BJG, 2 represents standard substance. **(A)** Mullein isoflavone glucoside. **(B)** Naringin. **(C)** Hesperidin. **(D)** Ononin. **(E)** Epimedoside A. **(F)** Icariin. **(G)** Nobiletin. **(H)** Tangeretin. **(I)** honokiol.

# 2 Determination of 9 chemical constituents in BJG by HPLC

# 2.1 Sample preparation

10.0 g powder of BJG was accurately weighed and combined with 25 ml of methanol and ultrasonically extracted for 30 min, and then filtered.

# 2.2 Elution gradient optimizing

# An Agilent 1260 infinity Ⅱ HPLC system was used (Agilent Technologies, Santa Clara, CA, USA). Agilent Poroshell 120 EC-C18 (100 mm×3.0 mm, 2.7 μm); mobile phase: 0.1% formic acid water (A)-acetonitrile (B); Gradient elution conditions: (1) 0-60 min, 3-100% B; (2) 0-60 min, 3-50% B; (3) 0-5 min, 3-3% B; 5-60 min, 3-48% B; detection wavelength: 230 nm; flow rate: 0.6 ml·min^-1^; column temperature: 40℃; injection volume: 5 μl.

**2.3 Detection wavelength optimizing**

# Agilent Poroshell 120 EC-C18 (100 mm×3.0 mm, 2.7 μm); mobile phase: 0.1% formic acid water (A)-acetonitrile (B); Gradient elution conditions: 0-5 min, 3-3% B; 5-60 min, 3-48% B; detection wavelength: 210, 230, 254, 280, 300, 320 nm; flow rate: 0.6 ml·min^-1^; column temperature: 40℃; injection volume: 5 μl.

**2.4 Results of elution gradient optimizing**

By investigating three different elution gradients, the optimal elution condition was determined as (3).

**
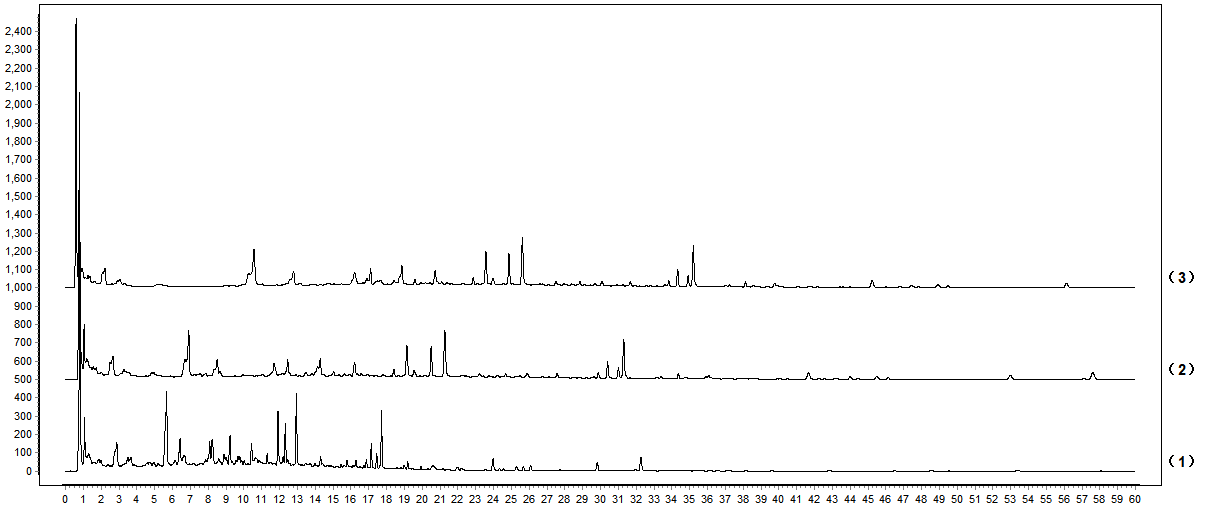
**

**Supplementary Figure 2.** Fingerprint chromatogram of BJG in different elution gradients. **(A)** Gradient elution conditions: 0-60 min, 3-100% B. **(B)** Gradient elution conditions: 0-60 min, 3-50% B. **(C)** Gradient elution conditions: 0-5 min, 3-3% B; 5-60 min, 3-48% B.

**2.5 Results of detection wavelength optimizing**

By investigating six different detection wavelengths, fingerprint chromatogram under 230 nm presents best: stable baseline, good separation, excellent response.

**
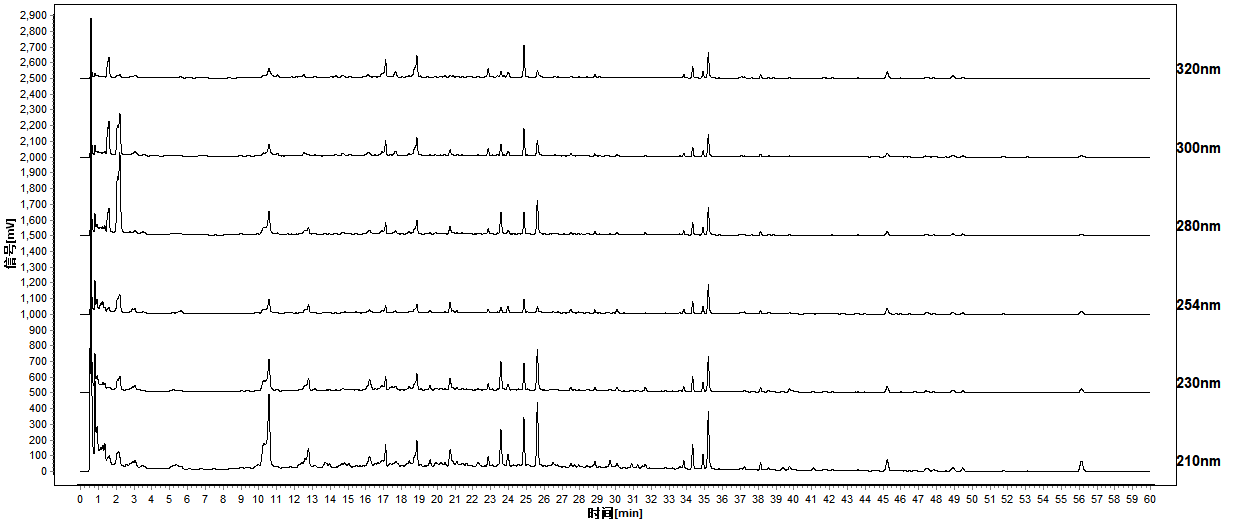
**

**Supplementary Figure 3.** Fingerprint chromatogram of BJG in different detection wavelengths. **(A)** 210 nm. **(B)** 230 nm. **(C)** 254 nm. **(D)** 280 nm. **(E)** 300 nm. **(F)** 320 nm.

**2.6 Determination of the content of 9 components**

The contents of mullein isoflavone glucoside, naringin, hesperidin, ononin, epimedoside A, icariin, nobiletin, tangeretin and honokiol were 0.071 mg/g, 0.172 mg/g, 0.028 mg/g, 0.017 mg/g, 0.021 mg/g, 0.343 mg/g, 0.047 mg/g, 0.018 mg/g and 0.042 mg/g respectively. The chromatogram is shown in Supplementary Figure 4.


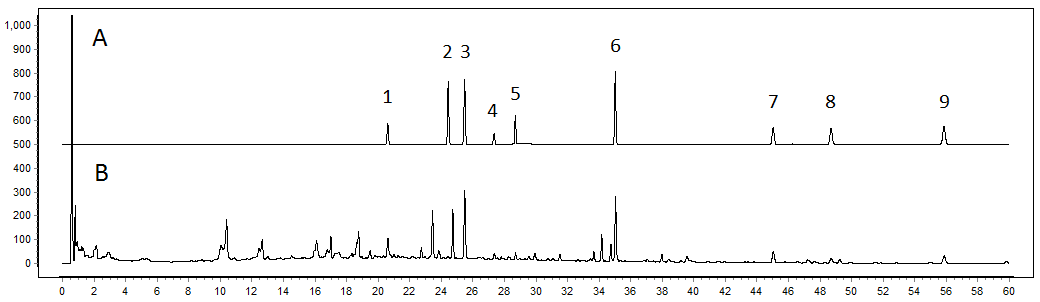


**Supplementary Figure 4.** Fingerprint chromatogram of BJG under 230nm. **(A)** Chromatogram of standard substances. 1-Mullein isoflavone glucoside. 2-Naringin. 3-Hesperidin. 4-Ononin. 5-Epimedoside A. 6-Icariin. 7-Nobiletin. 8-Tangeretin. 9-honokiol. **(B)** Chromatogram of sample.
